# Spatiotemporal dynamics of dendritic spines in the living brain

**DOI:** 10.3389/fnana.2014.00028

**Published:** 2014-05-09

**Authors:** Chia-Chien Chen, Ju Lu, Yi Zuo

**Affiliations:** ^1^Department of Molecular, Cell and Developmental Biology, University of California at Santa CruzSanta Cruz, CA, USA; ^2^Department of Biological Sciences and James H. Clark Center, Stanford UniversityStanford, CA, USA

**Keywords:** dendritic spine, *in vivo*, two-photon imaging, experience-dependent plasticity, neural circuit, cerebral cortex

## Abstract

Dendritic spines are ubiquitous postsynaptic sites of most excitatory synapses in the mammalian brain, and thus may serve as structural indicators of functional synapses. Recent works have suggested that neuronal coding of memories may be associated with rapid alterations in spine formation and elimination. Technological advances have enabled researchers to study spine dynamics *in vivo* during development as well as under various physiological and pathological conditions. We believe that better understanding of the spatiotemporal patterns of spine dynamics will help elucidate the principles of experience-dependent circuit modification and information processing in the living brain.

## INTRODUCTION

Dendritic spines have fascinated generations of neuroscientists since their initial description by Santiago Ramón y Cajal more than a century ago (Ramon y Cajal, 1888). These delicate protrusions emanate from the dendritic shaft and resemble “bristling thorns or short spines” as described vividly by Cajal. They are the postsynaptic sites of the great majority (>90%) of excitatory glutamatergic synapses in the mammalian brain, and contain essential molecular components for postsynaptic signaling and plasticity. Therefore, spines and their structural dynamics may serve as indicators for synaptic connectivity and modifications thereof (Segal, 2005; Tada and Sheng, 2006; Harms and Dunaevsky, 2007).

Most early studies on the dendritic spine examined fixed neural tissue with light or electron microscopy (Lund et al., 1977; Woolley et al., 1990; Harris and Kater, 1994; Hering and Sheng, 2001; Lippman and Dunaevsky, 2005). Although they provided fundamental information about spine morphology and distribution, these fixed tissue examinations only captured static “snapshots” of spines. The introduction of fluorescent labeling techniques and multi-photon microscopy revolutionized the field. In 2002, the pioneering work from two laboratories (Grutzendler et al., 2002; Trachtenberg et al., 2002) demonstrated the possibility to track the same spine in the living brain over a long period (i.e., weeks) of time. In principle, spine dynamics represent synapse dynamics. While stable spines mostly represent synaptic contacts, only a small fraction of transient spines represent short-lived synaptic contacts, and the rest of them represent failed synaptogenesis (Trachtenberg et al., 2002; Knott et al., 2006; Cane et al., 2014). From such time-lapse imaging studies a dynamic picture of spines has emerged: spines form, enlarge, shrink, and retract throughout the animal’s lifespan. Furthermore, their morphology and dynamics vary among neuronal types, across developmental stages, and in response to experiences such as sensory stimulation and deprivation, environmental enrichment, and various paradigms of learning (Holtmaat and Svoboda, 2009; Fu and Zuo, 2011).

This review focuses on results from *in vivo* imaging studies. In characterizing spine dynamics, researchers have mainly considered two aspects: overall changes in spine density, and the specific location along the dendrite where spine formation and elimination occur. While spine density provides an approximate estimate of the total number of excitatory synapses onto the postsynaptic neuron, the location of a spine influences the contribution of its synaptically transmitted electrical and chemical signals to the integrated response at the soma (Nevian et al., 2007; Spruston, 2008). Understanding how spine dynamics correlate with anatomical and physiological features of specific neural circuits in different behavioral contexts is crucial to the elucidation of the information processing and storage mechanisms in the brain.

## SPINE DYNAMICS DURING DEVELOPMENT

**FIGURE 1:**
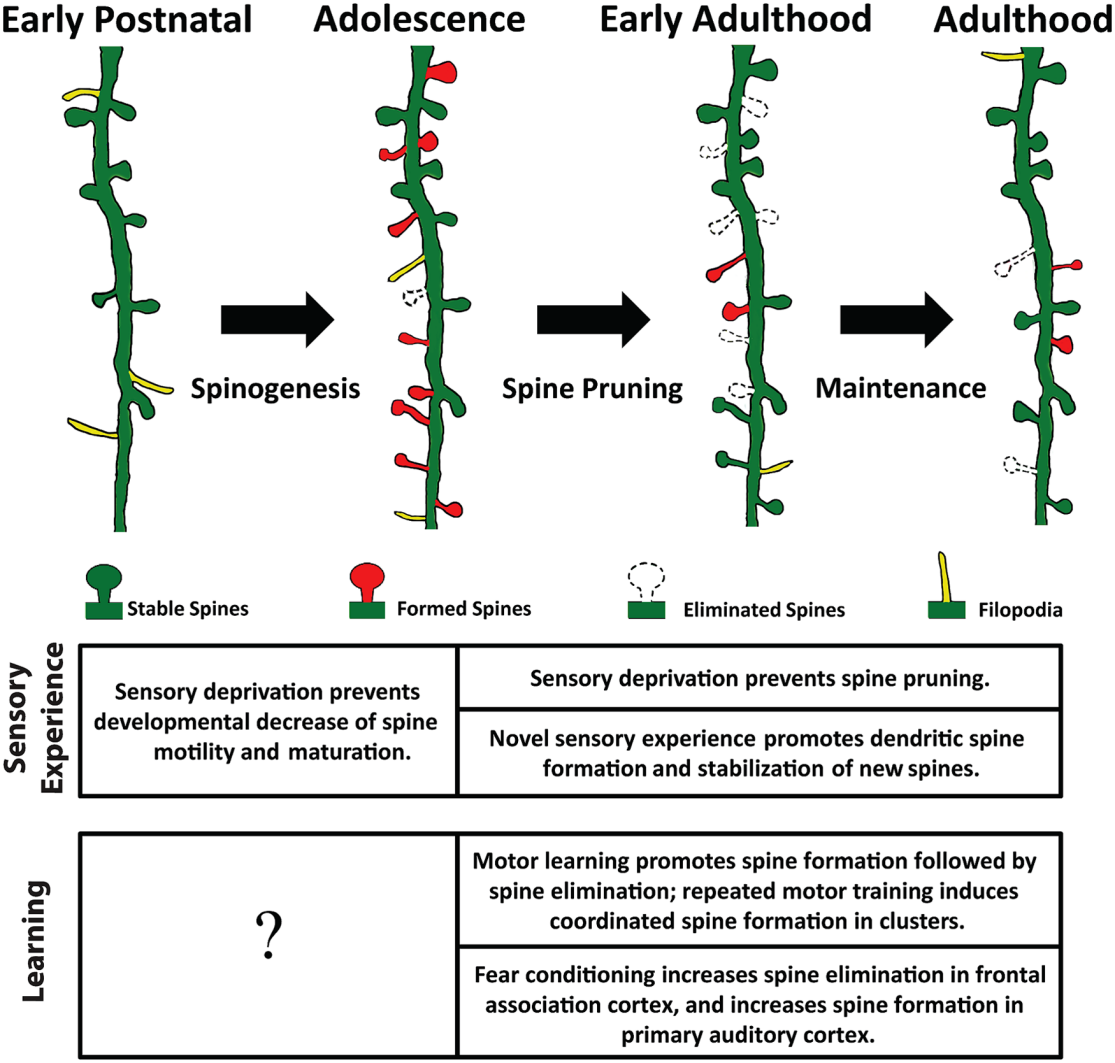
**Spine remodeling at different stages of an animal’s life**. Rapid spinogenesis in early postnatal is followed by a gradual spine pruning in adolescence. In adulthood, spine formation and elimination reach equilibrium, with a small fraction of spines constantly added or removed. Experience affects spine dynamics differently at different developmental stages.

Spine density varies significantly across diverse populations of neurons, likely reflecting the diversity of neuronal morphology and function (Nimchinsky et al., 2002; Ballesteros-Yanez et al., 2006). The balance between spine formation and elimination determines the change in spine density: a surplus of spine formation over elimination along a dendritic segment increases spine density thereon, and vice versa. In the cerebral cortex, while dendritic branches are mostly stable over time (Trachtenberg et al., 2002; Mizrahi and Katz, 2003; Chow et al., 2009; Mostany and Portera-Cailliau, 2011; Schubert et al., 2013), spines are constantly formed and eliminated. The rates of spine formation and elimination change over time, resulting in non-monotonic alteration in spine density (**Figure 1**). For example, spines on the apical dendrites of layer 2/3 pyramidal neurons in rodent barrel cortex exhibit gradually decreasing motility (elongation and shortening of spines) and turnover rate (defined as the total amount of gains and losses of spines) between postnatal day 7 and 24 (P7-24; Lendvai et al., 2000; Cruz-Martin et al., 2010). Nevertheless, spine density continuously increases over this period of time (Cruz-Martin et al., 2010). After this initial phase of net spine gain, spine elimination starts to outpace formation, leading to an overall reduction of spine density (Holtmaat et al., 2005; Zuo et al., 2005b; Yang et al., 2009). Between P28 and P42, 17% of spines are eliminated along the apical dendrites of layer 5 pyramidal neurons in the mouse barrel cortex, while only 5% of new spines are formed during the same period of time (Zuo et al., 2005a, b). Importantly, not all spines are equally susceptible to elimination: those with large heads are more stable than thin ones. As spine head size correlates with synaptic strength, this phenomenon suggests that stronger synapses are more stable (Holtmaat et al., 2005). Furthermore, newly formed spines are more likely to be eliminated than pre-existing spines (Xu et al., 2009), and the majority of stable spines formed before adolescence remain incorporated in the adult neuronal circuit (Zuo et al., 2005a; Yang et al., 2009; Yu et al., 2013). Finally, in adult animals spine formation and elimination reach equilibrium; spine density remains roughly constant until the onset of aging (Zuo et al., 2005a; Mostany et al., 2013).

## SPINE DYNAMICS IN RESPONSE TO SENSORY EXPERIENCE

The cerebral cortex has the amazing ability to reorganize its circuitry in response to experiences. Therefore, how sensory experiences (or lack thereof) impact spine dynamics is of great interest to neuroscientists. Both acute and chronic sensory manipulations have been shown to profoundly impact spine dynamics, but the exact effect depends on the manipulation paradigm and duration, as well as the developmental stage of the animal. During early postnatal period, sensory inputs play instructive roles in the stabilization and maturation of spines. In the mouse visual cortex, depriving visual input from birth prevented the decrease in spine motility and maturation of spine morphology (Majewska and Sur, 2003; Tropea et al., 2010). Genetic deletion of the PirB receptor mimicked the effect of monocular deprivation on spine motility (Djurisic et al., 2013). In mice that had been subjected to visual deprivation previously, light-induced spine maturation could be partially mimicked by pharmacological activation of the GABAergic system, suggesting an important role of inhibitory circuits in the maturation of excitatory synapses (Tropea et al., 2010). Later on, sensory experience drives spine pruning (defined as net loss of spines). Unilateral trimming of all whiskers in 1-month-old mice for 4 or 14 days dramatically reduced spine elimination in the barrel cortex, but left spine formation largely unperturbed (Zuo et al., 2005b; Yu et al., 2013). Pharmacological blockade of NMDA receptors mimicked the effect of whisker trimming, indicating the involvement of the NMDA receptor pathway in such activity-dependent spine elimination (Zuo et al., 2005b).

While complete whisker trimming removes sensory input globally, trimming every other whisker (“chessboard trimming”) presumably amplifies any difference in activity levels and patterns of neighboring barrels, thereby introducing a novel sensory experience. Such paradigm has been shown to promote spine turnover and to stabilize newly formed spines selectively in a subclass of cortical neurons (Trachtenberg et al., 2002; Holtmaat et al., 2006). New spines were preferentially added onto layer 5 pyramidal neurons with complex apical tufts, rather than those with simple tufts (Holtmaat et al., 2006). In αCaMKII-T286A defective mice, chessboard trimming failed to increase stabilization of new persistent spines at the border between spared and deprived barrels (Wilbrecht et al., 2010). Recently, an elegant study combining optogenetic stimulation and *in vivo* imaging showed that it is the pattern of neural activity, rather than the magnitude, that determines the stability of dendritic spines (Wyatt et al., 2012).

Similar to chessboard trimming, brief monocular deprivation (MD) increases the disparity between the inputs from two eyes. Thus similar to chessboard trimming, MD has been found to increase spine formation along apical dendritic tufts of layer 5 pyramidal neurons in the binocular zone of the mouse visual cortex. However this effect was not observed in layer 2/3 neurons, or in the monocular zone (Hofer et al., 2009), again indicating a cell type specific synapse remodeling. Interestingly, a second MD failed to increase spine formation further, but selectively enlarged the spines formed during the initial MD, suggesting that new spines formed during the initial MD had functional synapses that were reactivated during the second MD (Hofer et al., 2009).

## SPINE DYNAMICS DURING LEARNING

The highly dynamic nature of dendritic spines elicits the prevalent idea that spines may serve as the structural substrate for learning and memory. It has been suggested that newly emerged spines (typically with small heads) underlie memory acquisition, while stable spines (typically with large heads) serve as memory storage sites (Bourne and Harris, 2007). Indeed, *in vivo* imaging studies have shown that in the cerebral cortex, spine dynamics directly correlates with learning. In the mouse motor cortex, spine formation begins immediately as the animal learns a new task. Following this rapid spinogenesis, spine density reverts to the baseline level through elevated spine elimination (Xu et al., 2009; Yu and Zuo, 2011). In song birds, higher baseline spine turnover rate before song learning has been found to correlate with a greater capacity for subsequent song imitation (Roberts et al., 2010). In mice, the amount of spines gained during initial learning closely correlates with the motor performance of learning acquisition (Xu et al., 2009); and survival of new spines correlates with retention of the motor skill (Yang et al., 2009). Furthermore, different motor skills are likely encoded by different subpopulations of synapses in the motor cortex, as learning a novel motor task in pre-trained mice continues to induce robust turnover in the adult motor cortex (Xu et al., 2009). Recently, it has also been found that the glucocorticoid level impacts motor learning-induced spine dynamics. Training mice at glucocorticoid peaks resulted in higher rate of spine formation, whereas glucocorticoid troughs following training was necessary for stabilization of spines formed during training and long-term memory retention (Liston et al., 2013). Addiction, which has been considered as pathological learning (Hyman, 2005), elicits similar temporal changes in spine dynamics as motor learning does. Using a cocaine-conditioned place preference paradigm, a recent imaging study showed that initial cocaine exposure promoted spine formation in the frontal cortex, and that the amount of new persistent spines correlated with the preference for the cocaine-paired context (Munoz-Cuevas et al., 2013). More interestingly, spine dynamics in different cortical regions may vary during the same task. For example, a fear conditioning paradigm that pairs auditory cues with foot shocks has demonstrated opposite effects in auditory and frontal cortex. In the auditory cortex, it was found that increased spine formation was correlated with paired fear conditioning, while unpaired conditioning was associated with increased elimination of spines (Moczulska et al., 2013). In the frontal association cortex, increased spine elimination was found to be associated with learning, while spine formation was associated with fear extinction, and reconditioning eliminated spines formed during extinction (Lai et al., 2012). Taken together, these studies reveal the diversity of temporal rules underlying learning-induced spine dynamics. Whether spines are formed or removed during learning depends on the behavioral paradigm as well as the specific neuronal circuit and cell types participating in the learning process.

It is worth noting that all the examples discussed above refer to non-declarative memory, which does not involve the conscious recollection of specific time, location, and episodic experience (i.e., declarative memory). Exploration of *in vivo* spine dynamics associated with declarative memory proves to be much more challenging. On one hand, hippocampus, the structure crucial for formation of declarative memory, is buried beneath cortex and beyond the reach of standard two-photon microscopy. On the other hand, declarative memory is believed to be diffusely stored in the large neocortical networks, making it difficult for targeted imaging. Therefore, the advancement of deep brain imaging techniques (e.g., microendoscopy, adaptive optics) together with a better understanding of memory allocation in the cortex holds the key to future investigation of spine dynamics underlying declarative memory.

## SPINE DYNAMICS IN DISEASES

Alterations in dendritic spine densities have been observed in various neurological and neuropsychiatric diseases. Each disorder presents with its own hallmark abnormalities in spine dynamics, which further corroborates the idea that spines are structural underpinnings for proper cognitive functioning. There is growing consensus that spine abnormality is associated with behavioral deficiency and decline in cognitive functions (for detail see Fiala et al., 2002; Penzes et al., 2011).

In stroke models, it is shown that severe ischemia leads to rapid spine loss, which is reversible after reperfusion if the rescue is performed within a short period of time (20–60 min; Zhang et al., 2005). Following stroke, spine formation and subsequent elimination increase in the peri-infarct region, but not in cortical territories distant from the infarct or in the contralateral hemisphere (Brown et al., 2009; Johnston et al., 2013). This injury-induced plasticity reaches its peak at 1 week post-stroke; from then on the rate of spine formation and elimination steadily decline. This phenomenon suggests the existence of a critical period during which the surviving peri-infarct cortical tissues are most amenable to therapeutic interventions (Brown et al., 2007, 2009). In a mouse model for chronic pain, partial sciatic nerve ligation increases spine formation and elimination. Similar to the stroke model, elevation of spine formation rate precedes that of elimination, leading to an initial increase in spine density followed by its reduction. Such effects could be abolished by tetrodotoxin blockade, indicating that post-lesion spine remodeling is activity-dependent (Kim and Nabekura, 2011).

Altered spine dynamics has also been reported in animal models of degenerative diseases. For example, spine loss is accelerated in the vicinity of β-amyloid plaques in the cerebral cortex (Tsai et al., 2004; Spires et al., 2005). In an animal model of Huntington’s disease spine formation rate increases, but newly formed spines do not persist to be incorporated into the local circuitry, which results in a net decrease in spine density (Murmu et al., 2013). While neurodegenerative diseases are usually associated with net spine loss, neurodevelopmental disorders exhibit diverse spine phenotypes. In a mouse model of Fragile X syndrome, spines are more numerous, and a higher percentage of them appear immature upon examination of adult fixed tissues (Comery et al., 1997; Irwin et al., 2000). *In vivo* studies further showed that in such animals spine turnover increased in various cortical areas (Cruz-Martin et al., 2010; Pan et al., 2010; Padmashri et al., 2013), and neither whisker trimming nor motor learning could further alter spine dynamics (Pan et al., 2010; Padmashri et al., 2013). In mice overexpressing MECP2, a Rett Syndrome related gene, it has been found that both spine gains and losses are elevated. However, new spines are more vulnerable to elimination than in wild type mice, resulting in a net loss of spines (Jiang et al., 2013).

## GLIAL CONTRIBUTION TO SPINE DYNAMICS

The nervous system comprises two classes of cells: neurons and glia. The most intriguing role of glial cells is their participation at synaptic functioning and dynamics. Recently, a few exciting studies explored the role of glial signaling in spine maturation and plasticity. For example, blockade of astrocytic glutamate uptake has been shown to accelerate experience-dependent spine elimination during adolescent development (Yu et al., 2013). Another type of glial cells, microglia, have also been found to be in close contact with dendritic spines. The motility of microglial processes and spine contact are actively regulated by sensory experience and are involved in spine elimination (Tremblay et al., 2010). In addition, the depletion of microglia resulted in significant reduction of motor learning induced spine formation, and selective removal of brain-derived neurotrophic factor (BDNF) in microglia recapitulated the effects of microglial depletion (Parkhurst et al., 2013).

## SPATIAL MANIFESTATION OF SPINE DYNAMICS

Structural imaging of spines has suggested that the emergence and disappearance of spines are neither uniform nor random along dendrites, but rather occur at spatially selective “hot spots.” In the mouse motor cortex, new spines that form during repeated training with the same motor task tend to cluster. Furthermore, addition of the second new spine in the cluster is often associated with the enlargement of the first new spine. In contrast, spines formed during tandem execution of different motor tasks or during motor enrichment do not cluster (Fu et al., 2012). Taken together, these observations suggest that repeated re-activation of the first new spine is required for the clustered emergence of the second new spine. Similar spatial selectivity of spine dynamics has been observed in the fear conditioning paradigm: a spine eliminated during fear conditioning is usually replaced by a spine in its vicinity (within 2 μm) during fear extinction (Lai et al., 2012). Interestingly, spine dynamics are also influenced by dynamics of inhibitory synapses. Monocular deprivation significantly increases coordinated dynamics of spines and the inhibitory synapses nearby in layer 2/3 pyramidal neurons (Chen et al., 2012). These findings support the clustered plasticity model, which postulates that clustered synapses are more likely to participate in encoding the same information than synapses dispersed throughout the dendritic arbor (Govindarajan et al., 2006).

Combining *in vivo* whole-cell patch recording and single spine calcium imaging, a recent work has shown that spines tuned for different peak frequencies are interspersed along dendrites of pyramidal neurons in the mouse auditory cortex (Chen et al., 2011). This finding raises an interesting question: do clustered new spines correspond to inputs with similar or different characteristics (e.g., activity patterns, tuning properties)? In order to address this question, it will be necessary to sample spines over a broad area of the dendritic arbor, identify “hotspots” of spine remodeling, and combine structural imaging of spines with real-time functional imaging. Such experiments will not only help elucidate the cellular mechanisms of activity-dependent spine remodeling, but also provide clues to information representation and storage in neurons.

## FUTURE DIRECTIONS

In this article, we have reviewed recent investigations on the dynamics of dendritic spines in the living brain. Although these studies have significantly advanced our understanding of how spine dynamics alter temporally and spatially, many questions remain on various fronts. For example, are there molecular markers that distinguish stable spines from newly formed spines and spines to be eliminated? Is the total number of spines maintained through a homeostatic mechanism, so that the dendrite may sustain the metabolic demand of synaptic transmission? Does clustering of new spines reflect changes in the strength of existing connections with the same axon (while maintaining the same network topology), or does it indicate the establishment of additional connections with previously unconnected axons nearby? It is worth noting that all works discussed above have focused on the postsynaptic side, which is only half of the story. The other major determinant of spine distribution and dynamics lies at the presynaptic side: the identity and geometry of presynaptic axons and the availability of axonal boutons. Knowing such presynaptic information is crucial in resolving many of the questions arising from observations of spine dynamics. However, identification of the presynaptic partner of an imaged dendritic spine remains a technical challenge, as the presynaptic axon may originate from a plethora of sources, and is usually intermingled with many other axonal processes. In addition, much remains to be learned about the sequence of structural remodeling that occurs at the contact site between the axonal bouton and the spine, and how such sequence associates with formation and elimination of synapses. Simultaneous imaging of axonal boutons and their partnering spines in the context of behavioral manipulation will provide abundant information to address this question. Retrospective ultrastructural examinations such as electron microscopy (Knott et al., 2009) and Array Tomography (Micheva and Smith, 2007; Micheva et al., 2010) may also complement *in vivo* imaging to validate the presence of synapses, and to reveal molecular fingerprints of imaged structures.

The temporal sequence and spatially selective rearrangements of neuronal connections, and how these changes collectively contribute to alterations of behavior as the result of experiences, is one of the fundamental questions in neuroscience. Advancement in imaging techniques, together with development in electrophysiology, molecular genetics and optogenetics, will help reveal the blueprint of neuronal circuitry at the microscopic level, as well as the mechanisms of information encoding, integration and storage in the brain.

## AUTHOR CONTRIBUTIONS

Chia-Chien Chen made the figure. Chia-Chien Chen, Ju Lu, and Yi Zuo wrote the manuscript.

## Conflict of Interest Statement

The authors declare that the research was conducted in the absence of any commercial or financial relationships that could be construed as a potential conflict of interest.
